# Draft Genome Sequence of the Polychlorinated Biphenyl-Degrading Bacterium *Pseudomonas stutzeri* KF716 (NBRC 110668)

**DOI:** 10.1128/genomeA.01215-15

**Published:** 2015-10-22

**Authors:** Jun Hirose, Atsushi Yamazoe, Akira Hosoyama, Nobutada Kimura, Hikaru Suenaga, Takahito Watanabe, Hidehiko Fujihara, Taiki Futagami, Masatoshi Goto, Kensuke Furukawa

**Affiliations:** a Department of Applied Chemistry, Faculty of Engineering, University of Miyazaki, Miyazaki, Japan; b Biological Resource Center, National Institute of Technology and Evaluation (NITE), Tokyo, Japan; c Bioproduction Research Institute, National Institute of Advanced Industrial Science and Technology (AIST), Tsukuba, Japan; d Research Institute for Sustainable Humanosphere, Kyoto University, Uji, Kyoto, Japan; e Department of Food and Nutrition, Beppu University, Beppu, Japan; f Education and Research Center for Fermentation Studies, Faculty of Agriculture, Kagoshima University, Kagoshima, Japan; g Laboratory of Future Creation Microbiology, Faculty of Agriculture, Kyushu University, Fukuoka, Japan

## Abstract

*Pseudomonas* stutzeri KF716 (NBRC 110668) utilizes biphenyl as a sole source of carbon and energy and degrades polychlorinated biphenyls. Here, we report the first draft genome sequence of a biphenyl-degrading strain of the species *P. stutzeri*.

## GENOME ANNOUNCEMENT

Polychlorinated biphenyls (PCBs) have become serious and global environmental contaminants because these compounds were widely used for a variety of industrial purposes due to their chemical and physical stabilities. The biphenyl-utilizing bacteria cometabolize certain PCBs into chlorobenzoic acids using biphenyl-catabolic enzymes. However, the biodegradability of PCBs is highly dependent on chlorine substitutions, and the degradation capabilities of PCBs are strain-dependent. *Pseudomonas stutzeri* KF716 was isolated together with more than 10 bacterial strains (KF strains) from the soil near a biphenyl manufacturing plant in Kitakyushu, Japan, by enrichment culture with biphenyl (1). A 90-kb DNA region containing both the biphenyl/PCB catabolic *bph* gens and salicylate catabolic *sal* genes (termed the *bph-sal* element) was isolated from the *Pseudomonas putida* KF715 genomic cosmid libraries (2). The *bph-sal* element can be highly transferred by conjugation to various *Pseudomonas putida* strains (1, 2). Therefore, these KF strains could be adequate materials to clarify the molecular mechanisms of adaptive evolution for xenobiotics. We recently began working on the whole-genome sequencing of a series of KF strains (3–9) Here, we present the genomic feature of *P. stutzeri* KF716.

The draft genome sequence was determined by the National Institute of Technology and Evaluation (NITE) using a strategy combining the 454 GS FLX+ system (Roche) and the MiSeq paired-end sequencing system (Illumina). The reads obtained by the two systems were assembled using Newbler version 2.6 (Roche). The assembled genome is composed of 30 contigs (>913 bp) totaling 4,188,013 bp, with a G+C content of 64.2%. The *N*_50_ contig size and the largest contig size are 370,448 bp and 655,983 bp, respectively.

The draft genome sequence of the KF716 strain was annotated using RAST version 2.0 (10) and contains 5,792 predicted coding DNA sequences (CDSs), 3 rRNAs (5S, 16S and 23S), and 50 tRNA sequences. The coding sequences were classified into 2,965 subsystems, the most abundant of which were metabolism of amino acids derivatives (*n* = 314 CDSs) and carbohydrates (*n* = 273); cofactors, vitamins, prosthetic groups, and pigments (*n* = 258); and protein metabolism (*n* = 250). A comparison of genome sequences available in the RAST data sets revealed that *P. stutzeri* A1501 (11) is the closest neighbor of the KF716 strain with a score of 534, followed by *P. stuzeri* XLDN-R with a score of 462 (12). The *bph-sal* element, similar to that of *P. putida* KF715 identified previously (2), was found in a single contig. These data suggest that the *bph-sal* element is mobilizable between the KF strains in the environment and contributes to their genome evolution.

### Nucleotide sequence accession numbers.

The draft genome sequence of *P. stutzeri* KF716 has been deposited in DDBJ/EMBL/GenBank under the accession numbers BBQQ01000001 to BBQQ01000030.
